# FGD5-AS1 is an oncogenic lncRNA in pancreatic cancer and regulates the Wnt/β-catenin signaling pathway via miR-577

**DOI:** 10.3892/or.2021.8232

**Published:** 2021-11-24

**Authors:** Wei-Tao Zhang, Ji-Jun Zhang, Quan Shao, Ying-Kai Wang, Jie-Peng Jia, Bo Qian, Xiao-Wen Tian, Wen-Ji Yan

**Affiliations:** 1 Cancer Center, Beijing Tongren Hospital, Capital Medical University, Beijing 100730, P.R. China; 2 Department of General Surgery, Sixth Hospital of Shanxi Medical University, Taiyuan, Shanxi 030008, P.R. China; 3 Department of Oncology, First Medical Center, Chinese PLA General Hospital, Beijing 100853, P.R. China

**Keywords:** FGD5-AS1, microRNA-577, LRP6, wnt/β-catenin signaling, pancreatic cancer

## Abstract

The objective of the present study was to clarify the expression characteristics of long non-coding RNA (lncRNA) FGD5 antisense RNA 1 (FGD5-AS1) in pancreatic cancer, as well as its biological function and underlying mechanism. Reverse transcription-quantitative polymerase chain reaction (RT-qPCR) was utilized for the detection of FGD5-AS1 and microRNA (miR)-577 expression levels in pancreatic cancer tissues. Transfection was performed to upregulate or downregulate FGD5-AS1 in pancreatic cancer cell lines. MTT and Transwell assays were then utilized to detect the proliferation, migration and invasion of cancer cells, respectively. Subsequently, dual-luciferase reporter gene assay, RNA immunoprecipitation assay, RNA pull-down assay, RT-qPCR, western blotting, and Pearson's correlation analysis were employed to confirm the regulatory relationships among FGD5-AS1, miR-577, low-density lipoprotein receptor-related protein 6 (LRP6) and β-catenin. Western blotting was employed to determine the expression levels of Axin2, cyclin D1 and c-Myc. The expression level of FGD5-AS1 was upregulated in pancreatic cancer tissues and cell lines. FGD5-AS1 knockdown inhibited pancreatic cancer cell proliferation, migration and invasion. By contrast, miR-577 was significantly inhibited in pancreatic cancer cells and tissues; its downregulation promoted pancreatic cancer cell proliferation, migration and invasion, and reversed the effects of FGD5-AS1 knockdown on pancreatic cancer cells. In addition, it was revealed that miR-577 was a target of FGD5-AS1, and FGD5-AS1 could modulate the expression levels of LRP6, β-catenin, Axin2, cyclin D1 and c-Myc via suppressing miR-577. In conclusion, in pancreatic cancer, highly expressed FGD5-AS1 activated the Wnt/β-catenin signaling and promoted cancer cell proliferation, migration and invasion via suppression of miR-577.

## Introduction

Pancreatic cancer is one of deadliest cancers (1). According to GLOBOCAN 2020 estimates, there were 495,773 new cases of pancreatic cancer and 466,003 deaths in 2020 (2). Due to insidious onset, special anatomical position, low resection rate and high recurrence rate, the 5-year survival rate of patients is <8% (3–5). Therefore, it is imperative to clarify the mechanism of pancreatic cancer progression in order to identify potential therapeutic targets and thus improve the treatment efficacy and survival rate of the patients.

It is reported that, 80% of DNA sequences could be transcribed into RNA, yet merely <2% of RNAs are translated into proteins; RNAs which have no protein-coding function are defined as non-coding RNAs (ncRNAs) (6). Long non-coding RNAs (lncRNAs) belong to ncRNAs, with a transcript longer than 200 nucleotides (7). Previously, lncRNAs were considered as ‘junk’. However, in previous years, growing studies have indicated that lncRNAs have crucial functions in biological processes (8,9). Since there are numerous lncRNAs specifically expressed or dysregulated in multiple cancers, lncRNAs are regarded as promising diagnostic biomarkers and treatment targets for cancer. LncRNA FGD5 antisense RNA 1 (FGD5-AS1) has been reported to be involved in the pathogenesis of acute myocardial infarction and periodontitis (10,11). Additionally, FGD5-AS1 is involved in the progression of clear renal cell carcinoma, colorectal cancer, and non-small cell lung carcinoma (12–15). Nonetheless, the role of FGD5-AS1 in pancreatic cancer has not been clarified.

Recognized as a highly conserved signaling pathway, Wnt/β-catenin is crucial in the development, organogenesis, tissue regeneration and other physiological or pathological processes (16). In cancer biology, the activation of the Wnt/β-catenin signaling is pivotal in regulating the sustaining proliferation, metastasis and chemoresistance of cancer cells (17). In the present study, the expression characteristics, biological functions and mechanism of FGD5-AS1 in pancreatic cancer were explored. It was revealed that FGD5-AS1 was highly expressed in pancreatic cancer tissues, and its overexpression facilitated the activation of the Wnt/β-catenin pathway and cancer progression via suppression of miR-577. The present study identified FGD5-AS1 as a novel carcinogenic lncRNA in pancreatic cancer, and partly explained the mechanism of the Wnt/β-catenin pathway dysregulation in pancreatic cancer.

## Materials and methods

### Clinical samples and ethical statement

The collection and use of human samples was carried out strictly following The Declaration of Helsinki (7th revision, 2008). The present study obtained approval from the Ethics Committee of the Sixth Hospital of Shanxi Medical University (approval no. 201310012; Taiyuan, China). The pancreatic cancer tissue and adjacent non-tumorous tissue samples were collected from 37 patients (21–78 years old; 21 males and 16 females) who had undergone surgery at the Sixth Hospital of Shanxi Medical University from January 2014 to June 2018. Patients who had no other digestive system tumors or tumor history, had never received any chemotherapy or radiotherapy, and were confirmed as pancreatic cancer by pathology and genetics were included. Patients with serious injury to the heart, liver, kidney and other important organs, history of autoimmune diseases, and chronic or acute infectious diseases were excluded. All of the patients had provided a written informed consent prior to inclusion in the study.

### Bioinformatics analysis

The expression pattern of FGD5-AS1 in pancreatic cancer tissues and normal tissues was predicted using the Gene Expression Profiling Interactive Analysis (GEPIA) database (http://gepia.cancer-pku.cn/). The potential downstream target miRNAs of FGD5-AS1 were predicted by the starBase database (http://starbase.sysu.edu.cn/panCancer.php) and LncBase Predicted v.2 database (http://carolina.imis.athena-innovation.gr) (18).

### Cell culture

Normal pancreatic ductal epithelial cells (HPDE6-C7 cells) and human pancreatic cancer cell lines (PANC-1, BXPC-3, CAPAN-1 and SW1990) were obtained from Shanghai Institute of Biochemistry and Cell Biology (Shanghai, China). Cells were cultured in Dulbecco's modified Eagle's medium (DMEM; Invitrogen; Thermo Fisher Scientific, Inc.) containing 0.1 mg/ml streptomycin, 100 U/ml penicillin and 10% fetal bovine serum (FBS; all from Hyclone; Cytiva) in an incubator at 37°C in 5% CO_2_.

### Cell transfection

Control siRNA (si-NC), FGD5-AS1 siRNA (si-FGD5-AS1), miR-577 mimics (miR-577), miR-577 inhibitors (miR-577 in), microRNA mimics control (miR-con), and microRNA inhibitor control (miR-con in) were obtained from Shanghai GenePharma Co., Ltd. Cells were cultured in 6-well plates, and when cell confluence reached 80–90%, the pancreatic cancer cells were cultured in the fresh serum-free and antibiotic-free medium for 8 h. Subsequently, 100 nM siRNAs or 50 nM miRNAs were transfected with pancreatic cancer cells using Lipofectamine^®^ 2000 (Invitrogen, Thermo Fisher Scientific, Inc.) at room temperature according to the supplier's protocol. A total of 12 h following transfection, the medium was replaced by complete medium, and the cell culture was continued for 24 h. Subsequently, the transfection efficiency was evaluated by reverse transcription-quantitative polymerase chain reaction (RT-qPCR). The sequences of the oligonucleotides were as follows: FGD5-AS1 siRNA1: 5′-UUGGUCGUUGUCAACUUCCCA-3′; FGD5-AS1 siRNA2: 5′-UAUUGUAUGAAUACACUGCUA-3′; si-NC: 5′-UUCUCCGAACGUGUCACGUTT-3′; miR-577 mimics: 3′-UAGAUAAAAUAUUGGUACCUG-5′; microRNA mimics control: 5′- UAAGUAGUCUGAAAUAGUUAC-3′; miR-577 inhibitors: 3′-CAGGUACCAAUAUUUUAUCUA-5′. microRNA inhibitor control: 5′-AUCAGUUCAAUCAUGUAUCAU-3′.

### RT-qPCR

Total RNA was extracted using TRIzol reagent (Invitrogen, Thermo Fisher Scientific, Inc.) following the manufacturer's protocol. To remove the DNA, 2 µg of RNA was treated with DNase and then reverse-transcribed into cDNA using SuperScript First Strand cDNA System (Invitrogen; Thermo Fisher Scientific, Inc.) according to the manufacturer's protocol. Subsequently, with cDNA as the template, RT-qPCR was performed using an ABI 7500 Fast Real-Time PCR System with SYBR^®^ PremixExTaq™ kit (Takara Biotechnology Co., Ltd.). The qPCR thermocycling conditions were as follows: pre-denaturation at 95°C for 10 min; followed by 40 cycles of denaturation at 95°C for 15 sec, and annealing and extension at 60°C for 60 sec. The primers were designed and synthesized by BGI (Shenzhen, China). The expression levels of FGD5-AS1, miR-577, low-density lipoprotein receptor-related protein 6 (LRP6) and β-catenin were determined with 2^−∆∆Cq^ method (19). The levels of U6 were used to normalize the miR-577 expression, and the levels of glyceraldehyde-3-phosphate dehydrogenase (GAPDH) were used to normalize the expression levels of lncRNA and mRNA. The sequences of the primers are listed in Table I.

### MTT assay

An MTT assay was utilized to determine cell proliferation rates. Cells in each group were transferred into 96-well plates (5×10^4^ cells/well), and routinely cultured. Following 12, 24, 48 and 72 h of culture, the cells were incubated with MTT reagent (Beyotime Institute of Biotechnology) for 4 h at 37°C. Subsequently, the formazan crystals were dissolved in dimethyl sulfoxide. Finally, a spectrophotometer was utilized to measure the absorbance of the cells at 570 nm.

### Transwell assay

In the migration assay, SW1990 cells were harvested and resuspended with serum-free medium, and then 200 µl of cell suspension (containing ~5×10^4^ cells) was added into the upper compartment of each Transwell chamber (8-µm pore size; BD Biosciences) in 24-well plates, and complete DMEM (containing glucose, L-glutamine, and sodium bicarbonate) with 10% FBS (600 µl per well) were added in the lower compartment. The cells were cultured for 24 h at 37°C, and then the cells on the upper surface of the membrane were gently wiped off with cotton swabs. The cells on the lower surface were fixed in 10% formalin for 20 min at room temperature and then stained with 0.1% crystal violet solution for 20 min at room temperature. Subsequently, the cells were counted under a light microscope. In the invasion assay, the procedures were the same as the migration assay using Transwell chambers covered with a layer of Matrigel (BD Biosciences) at 4°C overnight.

### Dual-luciferase reporter gene assay

To determine the binding association between FGD5-AS1 and miR-577, the wild-type (WT) or mutant (MUT) FGD5-AS1 sequence was subcloned into the psi-CHECK2 reporter vector (Promega Corporation). The cells were subsequently transferred to 24-well plates. A total of 24 h later, miR-577 mimics or miR-NC were co-transfected into SW1990 cells with WT or MUT FGD5-AS1 reporter vectors, respectively, using Lipofectamine 2000 (Invitrogen; Thermo Fisher Scientific, Inc.). Luciferase activity was determined 48 h following transfection. Luciferase activity was determined by a Dual-Luciferase^®^ Reporter Assay system (Promega Corporation). *Renilla* luciferase activity was used as a control for firefly luciferase activity. Similarly, the targeting relationship between miR-577 and LRP6 (or β-catenin) was determined. To determine the activity of Wnt/β-catenin pathway, the TOPflash and FOPflash system (Upstate Biotechnology, Inc.) was used. This system was used to evaluate β-catenin-dependent signaling which drives the expression of T-cell factor (TCF). TOPflash is a TCF reporter plasmid containing WT TCF binding sites driven by the thymidine kinase minimal prompter and an upstream luciferase reporter gene. FOPflash contains mutated TCF binding sites driven by the same thymidine kinase promoter and upstream luciferase reporter gene. FOPflash was used as a control for TOPflash activity.

### RNA immunoprecipitation (RIP) assay

The interaction between FGD5-AS1 and miR-577 was analyzed using the EZ-Magna RNA binding protein immunoprecipitation kit (cat. no. 17–700; EMD Millipore). Briefly, following transfection, 1×10^6^ SW1990 cells were lysed in the RIP lysis buffer (included in the kit), and then 100 µl of cell lysate was mixed with 50 µl of magnetic beads coupled with 5 µg of anti-Ago2 antibody (cat. no. MABE253; EMD Millipore) or anti-IgG antibody (cat. no. AP101; EMD Millipore) in RIP buffer. Following incubation at 4°C for 8 h, the sample was centrifuged at 12,000 × g for 30 sec at 4°C, and the supernatant was discarded, and then the immunoprecipitate was obtained and incubated at 55°C for 30 min with proteinase K (EMD Millipore) to remove the protein. The RNA was isolated using TRIzol reagent according to the manufacturer's protocol. Subsequently, the RNA was reversely transcribed into cDNA (the procedure was the same as aforementioned in RT-qPCR section). Finally, the level of FGD5-AS1 in the immunoprecipitate was analyzed by RT-qPCR.

### RNA pull-down assay

Biotin-labeled miR-577 (Bio-miR-577) and the control miRNAs (Bio-miR-NC) were incubated with 500 µl cell lysates at 4°C overnight. Then, streptavidin-coated 400 µl of magnetic beads (Thermo Fisher Scientific, Inc.) were added in the cell lysates, and incubated at 4°C overnight, to enrich the complex containing the biotin. Next, the complex was eluted, and then the RNA in the complex was extracted using TRIzol reagent as aforementioned. Subsequently, the FGD5-AS1 enrichment in the complex was evaluated via qPCR.

### Western blot assay

Cells were lysed in RIPA buffer (Pierce; Thermo Fisher Scientific, Inc.) containing protease inhibitors. Then the lysates were centrifuged at 12,000 × g for 15 min at 4°C and the supernatant was collected. The supernatant was added with loading buffer and boiled, and the protein samples were prepared. Next, 10% sodium dodecyl sulfate-polyacrylamide gel electrophoresis was employed to separate the protein samples (20 µg per lane), and then the proteins were electrotransferred onto polyvinylidene fluoride (PVDF) membranes (EMD Millipore). Subsequently, the proteins were blocked with 5% skimmed milk for 2 h at room temperature, and then incubated overnight with primary antibodies anti-Axin2 (product code ab109307; 1:1,000), anti-c-Myc (product code ab32072; 1:1,000), anti-cyclin D1 (product code ab16663; 1:1,000), β-actin (product code ab8227; 1:1,000; all from Abcam) at 4°C. After being rinsed three times with Tris-buffered saline and 0.05 % Tween-20 (TBST), the proteins were incubated with horseradish peroxidase-coupled secondary antibodies goat anti-rabbit IgG H&L (HRP) (ab205718; 1:5,000; Abcam) at room temperature for 30 min. Then, TBST was employed again, to wash the membranes 3 times. Ultimately, a hypersensitive ECL kit (Beyotime Institute of Biotechnology) was utilized to develop the protein bands on an X-ray film.

### Lung metastasis assay in vivo

A total of 20 nude mice (male; 4 weeks old; weighing ~16 g) were obtained commercially from the National Laboratory Animal Center (Beijing, China). Mice were housed and maintained at ~20°C in a relative humidity of 40–70% with a 12-h light/dark cycle and received food and water *ad libitum*. All the mice were randomly divided into two groups (si-NC group vs. si-FGD5-AS1 group, 10 mice per group). SW1990 cells were transfected with si-NC or FGD5-AS1 siRNA1. Then the cells were suspended in sterile phosphate-buffered saline and injected into the tail vein of each 4-week-old mouse (1×10^7^ cells/mouse) (20). The animal health and behaviour were monitored weekly. A total of 3 weeks later, all 20 mice were anesthetized by intraperitoneal injection with 10% chloral hydrate (300 mg/kg), and then sacrificed by decapitation. The lung tissues were fixed in 4% paraformaldehyde at 4°C for 24 h and embedded in paraffin; after which, 4-µm sections were cut and stained with hematoxylin for 5 min and eosin for 2 min at room temperature. Metastatic nodules in the lungs were evaluated by pathological examination under a light microscope (magnification, ×200). No mouse exhibited signs of peritonitis, pain or discomfort following the administration of 10% chloral hydrate. The animal experiments were approved (approval no. 8217113963) by the Institutional Animal Care and Use Committee of Chinese PLA General Hospital (Beijing, China).

### Statistical analysis

Data analysis was carried out using the SPSS 20.0 software (IBM Corp.). The data was presented as the mean ± standard deviation (SD). Pearson's correlation analysis was used to determine the correlation between the expression levels of FGD5-AS1 and miR-577 in pancreatic cancer tissues. The Kolmogorov-Smirnov test was used for normality and equal variance of the data. Unpaired Student's t-tests were utilized to perform comparisons. For data that had skewed distribution, comparisons between two groups were performed by Wilcoxon signed-rank test. P<0.05 was considered to indicate a statistically significant difference.

## Results

### FGD5-AS1 expression is increased in pancreatic cancer

**Figure 1.**
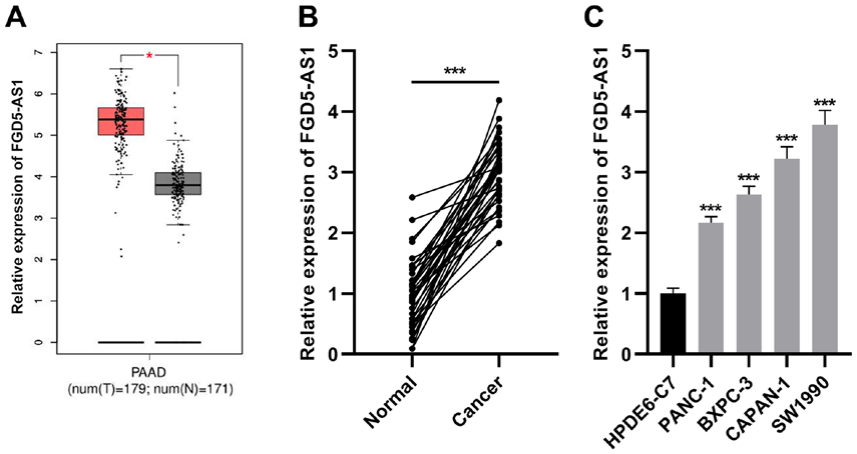
FGD5-AS1 expression is increased in pancreatic cancer. (A) Online bioinformatics analysis tool (GEPIA) was utilized for analyzing FGD5-AS1 expression in pancreatic cancer and normal tissues. (B) RT-qPCR was utilized for detecting FGD5-AS1 expression in collected pancreatic cancer tissues and adjacent tissue samples (n=37). (C) RT-qPCR was employed for detecting FGD5-AS1 expression in pancreatic cancer cell lines (SW1990, PANC-1, CAPAN-1 and BXPC-3) and normal pancreatic ductal epithelial cells (HPDE6-C7). All of the experiments were performed in triplicate. *P<0.05 and ***P<0.001. FGD5-AS1, FGD5 antisense RNA 1; RT-qPCR, reverse transcription-quantitative PCR.

GEPIA database (http://gepia.cancer-pku.cn/) revealed that FGD5-AS1 was differentially expressed in pancreatic cancer tissues and normal tissues, and FGD5-AS1 expression in cancerous tissues was higher than that in normal pancreatic tissues (Fig. 1A). Consistently, RT-qPCR revealed that FGD5-AS1 expression was significantly elevated in pancreatic cancer tissues in comparison to the adjacent non-tumorous tissues (Fig. 1B). Subsequently, RT-qPCR was utilized for detecting FGD5-AS1 expression in the pancreatic cancer cell lines (SW1990, PANC-1, BXPC-3 and CAPAN-1) and pancreatic ductal epithelial cell line (HPDE6-C7), and it was revealed that compared with HPDE6-C7 cells, FGD5-AS1 expression was increased in the pancreatic cancer cell lines (Fig. 1C).

### FGD5-AS1 knockdown suppresses SW1990 cell proliferation, migration and invasion

**Figure 2.**
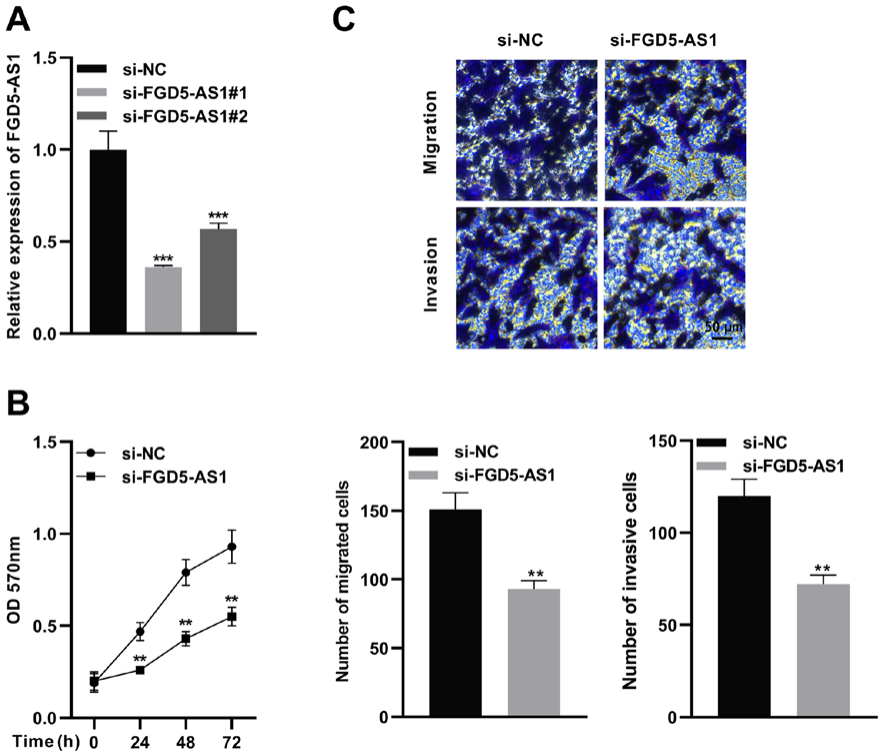
FGD5-AS1 knockdown suppresses pancreatic cancer cell proliferation, migration and invasion. (A) FGD5-AS1 siRNA or control siRNA was transfected into SW1990 cells to construct the knockdown model of FGD5-AS1, and RT-qPCR was employed to detect the interference efficiency of siRNAs. (B) An MTT assay was employed to detect the effect of knockdown of FGD5-AS1 on SW1990 cell proliferation. (C) Transwell assays were utilized to detect the effect of knockdown of FGD5-AS1 on SW1990 cell migration and invasion. Scale bar, 50 µm. All of the experiments were performed in triplicate. **P<0.01 and ***P<0.001. FGD5-AS1, FGD5 antisense RNA 1; si-, small interfering; RT-qPCR, reverse transcription-quantitative PCR; NC, negative control.

To determine the functions of FGD5-AS1 in pancreatic cancer progression, two siRNAs for FGD5-AS1 were designed. Then, FGD5-AS1 siRNA or control siRNA was transfected into SW1990 cells, and the knockdown efficiency was verified by RT-qPCR (Fig. 2A). Next, the more effective siRNA (si-FGD5-AS1 1) was selected for subsequent functional experiments. MTT and Transwell assays were utilized to detect proliferation, migration and invasion of SW1990 cells. It was revealed that FGD5-AS1 knockdown significantly suppressed viability, migration and invasion of SW1990 cells in contrast to the si-NC group (Fig. 2B and C).

### miR-577 is a downstream target of FGD5-AS1

**Figure 3.**
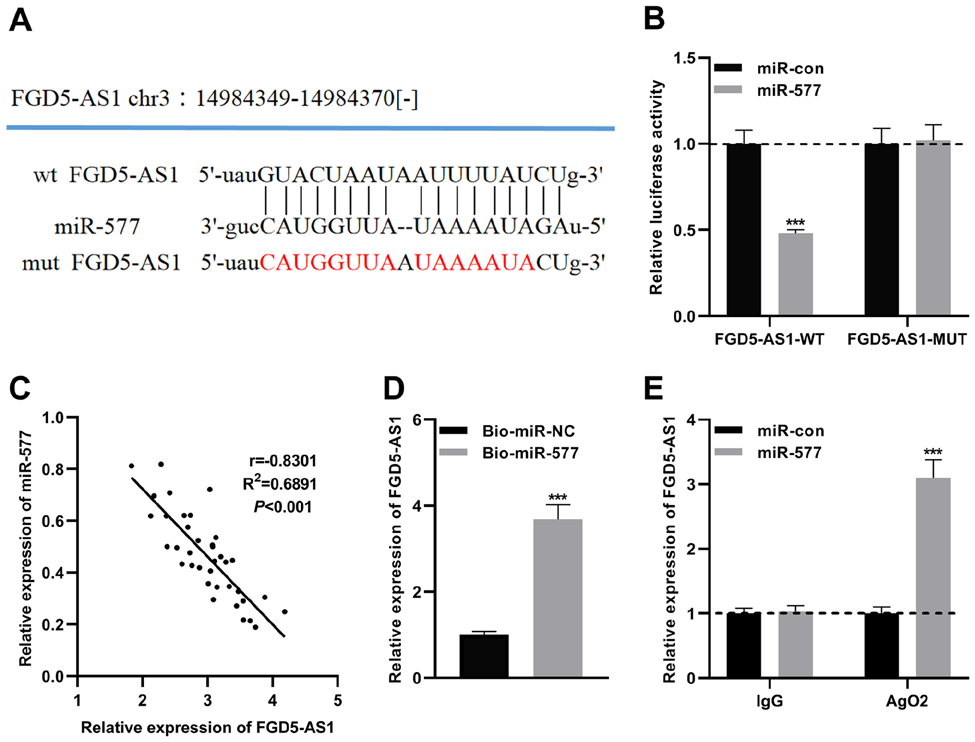
FGD5-AS1 directly targets miR-577. (A) Bioinformatics analysis was carried out to predict the binding sites between miR-577 and FGD5-AS1, and FGD5-AS1-WT and FGD5-AS1-MUT luciferase reporter gene vectors were constructed. (B) A dual-luciferase reporter assay was utilized to determine the binding site between FGD5-AS1 and miR-577. (C) Pearson's correlation analysis was employed to analyze the correlation between FGD5-AS1 expression and miR-577 expression in pancreatic cancer tissues. (D) An RNA pull-down assay was employed to validate the direct interaction between miR-577 and FGD5-AS1. (E) A RIP assay was carried out to confirm that the complex containing miR-577 and FGD5-AS1 in SW1990 cells was immunoprecipitated by anti-Ago2. All of the experiments were performed in triplicate. ***P<0.001. FGD5-AS1, FGD5 antisense RNA 1; miR, microRNA; WT, wild-type; MUT, mutant; RIP, RNA immunoprecipitation.

To clarify the mechanism of FGD5-AS1 in pancreatic cancer progression, the potential downstream target miRNAs of FGD5-AS1 were predicted by the starBase v2.0 database (http://starbase.sysu.edu.cn/panCancer.php) and LncBase Predicted v.2 database (http://carolina.imis.athena-innovation.gr) (Tables SI and SII). Notably, a potential binding site between miR-577 and FGD5-AS1 was predicted (Figs. 3A and S1). Recent research has revealed that miR-577 is downregulated in pancreatic ductal adenocarcinoma, and the low expression of miR-577 is significantly associated with poor prognosis (21). Therefore, the role of miR-577 in pancreatic cancer was investigated. To verify the targeting relationship between miR-577 and FGD5-AS1, MUT FGD5-AS1 luciferase reporter vector (FGD5-AS1-MUT) or WT FGD5-AS1 luciferase reporter vector (FGD5-AS1-WT) was co-transfected into SW1990 cells with miR-577 mimics or control miRNA, and it was revealed that miR-577 could only suppress the luciferase activity of FGD5-AS-WT (Figs. 3B and S2). Subsequently, Pearson's correlation analysis revealed that FGD5-AS1 and miR-577 expression levels were negatively correlated in pancreatic cancer tissues (Fig. 3C). Additionally, RNA pull-down and RIP assays were performed, and it was revealed that ectogenic miR-577 enriched FGD5-AS1, and miR-577 and FGD5-AS1 were enriched in Ago2-containing microribonucleoproteins (Fig. 3D and E). All of the aforementioned findings indicated that miR-577 is a direct downstream target of FGD5-AS1.

### Inhibition of FGD5-AS1 promotes SW1990 cell proliferation, invasion and migration

**Figure 4.**
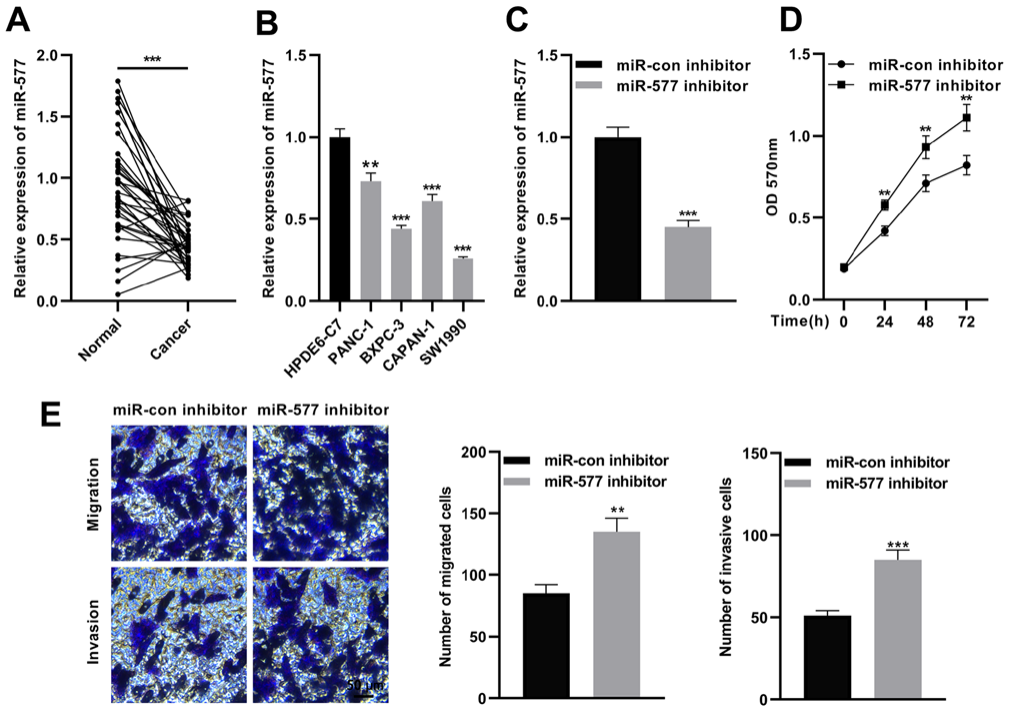
Inhibition of miR-577 facilitates pancreatic cancer cell proliferation, migration and invasion. (A) RT-qPCR was utilized to detect miR-577 expression in the pancreatic cancer tissue and adjacent tissue samples (n=37). (B) RT-qPCR was employed to detect miR-577 expression in pancreatic cancer cell lines (SW1990, PANC-1, CAPAN-1 and BXPC-3) and normal pancreatic ductal epithelial cells (HPDE6-C7). (C) SW1990 cells were transfected with miR-con inhibitors or miR-577 inhibitors to construct the knockdown expression model of miR-577, and the transfection efficiency was detected by RT-qPCR. (D) An MTT assay was employed to detect the effect of miR-577 inhibition on SW1990 cell proliferation. (E) Transwell assays were utilized to detect the effects of miR-577 inhibition on SW1990 cell migration and invasion. Scale bar, 50 µm. All of the experiments were performed in triplicate. **P<0.01 and ***P<0.001. miR, microRNA RT-qPCR, reverse transcription-quantitative PCR.

The expression of FGD5-AS1 in pancreatic cancer was then investigated. RT-qPCR revealed that miR-577 expression was significantly reduced in pancreatic cancer tissues in comparison with para-tumorous tissues (Fig. 4A), and compared with HPDE6-C7 cells, miR-577 expression was significantly decreased in pancreatic cancer cell lines (Fig. 4B). Next, miR-577 inhibitors or miR-con inhibitors were transfected into SW1990 cells (Fig. 4C). Subsequently, MTT and Transwell assays were used to detect proliferation, migration and invasion of SW1990 cells. As revealed in Fig. 4D and E, miR-577 inhibition significantly increased proliferation, migration and invasion of SW1990 cells. These results suggested that miR-577 is a tumor suppressor in pancreatic cancer.

### miR-577 regulates the Wnt/β-catenin signaling in pancreatic cancer via targeting LRP6 and β-catenin

**Figure 5.**
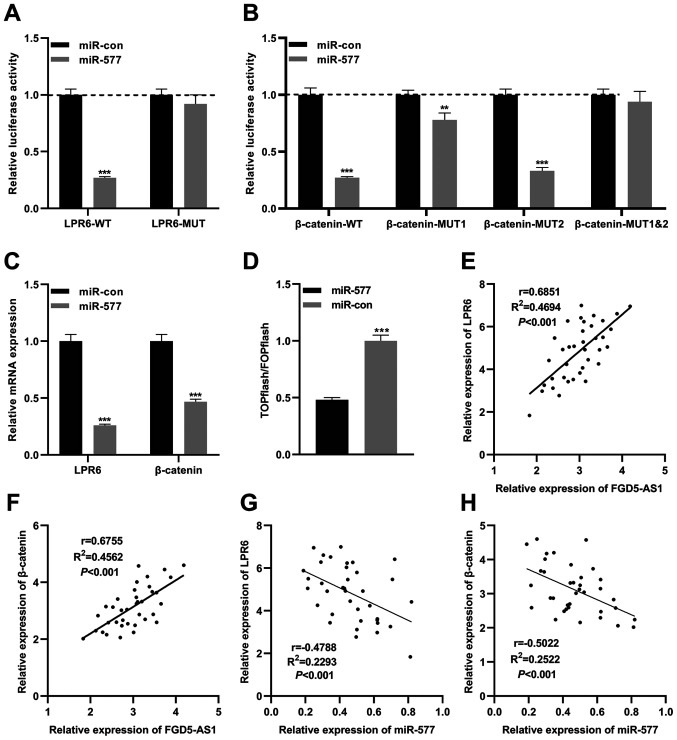
miR-577 targets LRP6 and β-catenin. (A) A dual-luciferase reporter assay was utilized to validate the binding sites between miR-577 and LRP6 3′UTR. (B) A dual-luciferase reporter assay was utilized to validate the binding sites between miR-577 and β-catenin 3′UTR. (C) Reverse transcription-quantitative PCR was employed to detect LRP6 and β-catenin mRNA expression levels in SW1990 cells transfected with miR-577 mimics. (D) A TOPFlash luciferase reporter assay was performed to detect the effects of miR-577 on the activity of the Wnt/β-catenin signaling. (E and F) Pearson's correlation analysis was utilized for the correlations between (E) FGD5-AS1 and LRP6 and between (F) FGD5-AS1 and β-catenin in pancreatic cancer tissues. (G and H) Pearson's correlation analysis was utilized for the correlations between (G) miR-577 and LRP6 and between (H) miR-577 and β-catenin in pancreatic cancer tissues. All of the experiments were performed in triplicate. **P<0.01 and ***P<0.001. LRP6, low-density lipoprotein receptor-related protein 6; miR, microRNA; FGD5-AS1, FGD5 antisense RNA 1; UTR, untranslated region.

A previous study reported that miR-577 inhibits the growth of glioblastoma multiforme via modulating the Wnt/β-catenin pathway; both β-catenin and LRP6, two crucial components of the Wnt/β-catenin signaling, are directly targeted by miR-577 (22). Therefore, in the present study, it was hypothesized that miR-577 was also associated with the Wnt/β-catenin signaling in pancreatic cancer. Based on the previous study (22), WT luciferase reporter vectors (LRP6-WT and β-catenin-WT) and MUT luciferase reporter vectors (LRP6-MUT, β-catenin-MUT1, β-catenin-MUT2 and β-catenin-MUT1&2) were constructed. Subsequently, reporter vectors and miR-577 mimics were co-transfected into SW1990 cells, and the dual-luciferase reporter assays were performed. As revealed in Fig. 5A, miR-577 mimics significantly reduced the luciferase activity of LRP6-WT, yet exerted no significant effect on that of LRP6-MUT. miR-577 significantly suppressed the luciferase activity of β-catenin-WT, β-catenin-MUT1 and β-catenin-MUT2, yet did not suppress that of β-catenin-MUT1&2 (Fig. 5B). The aforementioned data suggested that miR-577 could bind to LRP6 3′ untranslated region (UTR) and β-catenin 3′UTR in pancreatic cancer cells. As expected, following transfection of miR-577 mimics into SW1990 cells, RT-qPCR revealed that the expression levels of β-catenin mRNA and LRP6 mRNA were significantly reduced (Fig. 5C). Subsequently, miR-577 mimics (or control miRNA) and the Wnt/β-catenin signaling reporter vector TOPflash were co-transfected into SW1990 cells, and the luciferase activity was detected 2 days following transfection. It was revealed that miR-577 mimics notably decreased the luciferase activity of pancreatic cancer cells, while control miRNA exerted no significant effect, which suggested that miR-577 indeed suppressed the activity of the Wnt/β-catenin signaling (Fig. 5D). Furthermore, Pearson's correlation analysis revealed that FGD5-AS1 expression was positively correlated with LRP6 expression and β-catenin expression in pancreatic cancer tissues (Fig. 5E and F), whereas miR-577 expression was negatively correlated with them (Fig. 5G and H). These results indicated that LRP6 and β-catenin were directly negatively regulated by miR-577, and FGD5-AS1 could probably indirectly modulate the activity of the Wnt/β-catenin pathway.

### miR-577 reverses the effects of FGD5-AS1 on pancreatic cancer cells

**Figure 6.**
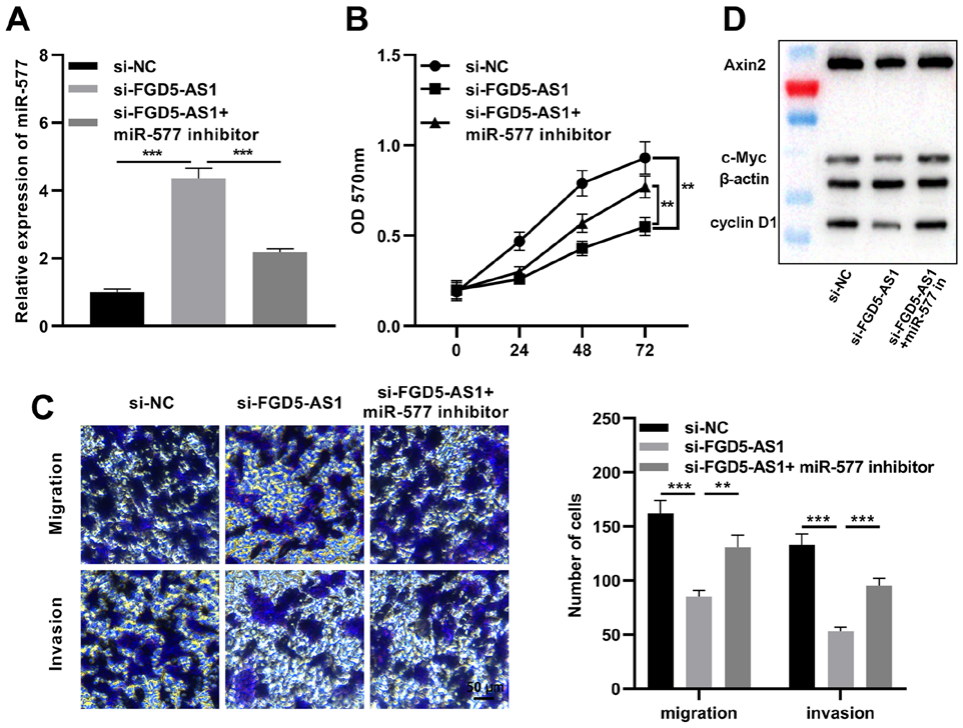
Inhibition of miR-577 partially reverses the biological behaviors of pancreatic cancer cells induced by FGD5-AS1 knockdown. (A) miR-577 inhibitors were transfected into SW1990 cells with FGD5-AS1 knockdown, and miR-577 expression in the transfected cells was detected by reverse transcription-quantitative PCR. (B) An MTT assay was utilized to detect the proliferation of SW1990 cells following transfection. (C) Transwell assays were utilized to detect the migration and invasion of SW1990 cells following transfection. Scale bar, 50 µm. (D) Western blotting was utilized to detect the protein expression of Axin2, cyclin D1 and c-Myc in SW1990 cells following transfection. All of the experiments were performed in triplicate. **P<0.01 and ***P<0.001. miR, microRNA; FGD5-AS1, FGD5 antisense RNA 1; si-, small interfering.

Subsequently, miR-577 inhibitors were transfected into SW1990 cells with FGD5-AS1 knockdown (Fig. 6A). The MTT assay revealed that miR-577 inhibition partially reversed the inhibitory effect of FGD5-AS1 knockdown on SW1990 cell proliferation (Fig. 6B). The Transwell assay indicated that miR-577 inhibition partially reversed the reduction caused by FGD5-AS1 knockdown in the migration and invasion of SW1990 cells (Fig. 6C). Additionally, to delve deeper into the association between the Wnt/β-catenin signaling pathway and the FGD5-AS1/miR-577 axis, western blotting was conducted to detect the expression levels of Axin2, cyclin D1 and c-Myc, which are considered as Wnt/β-catenin pathway-related proteins. It was revealed that FGD5-AS1 knockdown significantly decreased Axin2, cyclin D1 and c-Myc expression levels in SW1990 cells, and miR-577 inhibition partially counteracted these effects of FGD5-AS1 knockdown (Fig. 6D). The aforementioned findings suggested that FGD5-AS1 could regulate the activity of the Wnt/β-catenin signaling via modulating miR-577.

### FGD5-AS1 enhances the lung metastasis of pancreatic cancer cells in vivo

To confirm that FGD5-AS1 was indeed implicated in the metastasis of pancreatic cancer, a lung metastasis model was constructed with nude mice. After SW1990 cells were injected into the mice via the caudal vein, the metastatic nodules in the lung tissues were detected. It was revealed that SW1990 cells with FGD5-AS1 knockdown reduced metastatic potential (Fig. S3 and Table SIII), forming less metastatic nodules. This result further supported that FGD5-AS1 participates in the progression of pancreatic cancer.

## Discussion

It is estimated that there were approximately 44,330 mortalities caused by pancreatic cancer in the USA in 2018, and by 2030 it will be the second leading cause of cancer-related mortality in the USA (23,24). The anatomical position of pancreas is special, and the majority of patients with pancreatic cancer have already reached the advanced stage when they are diagnosed. In addition, the surgical resection rate is low and pancreatic cancer cells are not sensitive to chemotherapy (25). Therefore, the prognosis of pancreatic cancer is extremely poor. In recent years, molecular targeted therapy has been gradually applied to cancer treatment and has improved the prognosis of cancer patients (26). Therefore, it is of significant clinical value to explore the mechanism of pancreatic cancer as well as to explore novel therapeutic targets.

Multiple lncRNAs are aberrantly expressed in cancerous tissues and mediate cancer progression by regulating epigenetic modification, alternative splicing, transcription and protein translation and lncRNAs have drawn widespread attention as new therapeutic targets and biomarkers (27,28). In pancreatic cancer, lncRNAs play crucial roles in regulating various malignant biological behaviors of cancer cells, including proliferation, migration, invasion, epithelial-mesenchymal transition (EMT), chemoresistance and radioresistance (29). For example, lncRNA BX111 is overexpressed in pancreatic cancer tissues, and highly expressed BX111 is associated with advanced TNM stage, distant metastasis, lymphatic vessel invasion and short overall survival rate of patients; BX111 knockdown has been revealed to suppress pancreatic cancer cell growth, invasion and the EMT process (30). In pancreatic cancer, lncRNA HOTTIP is highly expressed, and its high expression is associated with shorter overall survival and disease-free survival (31). The role of FGD5-AS1 in cancer biology has gradually been revealed in recent years (12–15). The present study, for the first time, to the best of our knowledge, demonstrated that the expression level of FGD5-AS1 was enhanced in pancreatic cancer tissues and cells. In addition, it was revealed that FGD5-AS1 knockdown significantly suppressed the malignant phenotype of pancreatic cancer cells. Our demonstrations suggested that FGD5-AS1 is a potential biomarker and treatment target for pancreatic cancer.

miRNAs are involved in the tumorigenesis and progression of multiple malignancies including pancreatic cancer. miRNAs are non-coding RNAs with 21–25 nucleotides in length, which target the complementary sequences of mRNAs in the 3′-UTR to cause translation inhibition or mRNA degradation, thereby regulating pathological and physiological processes, such as cell proliferation, migration, differentiation, apoptosis and angiogenesis (32). Recent studies have reported that miR-577 is downregulated in multiple cancers, and its aberrant expression is related to tumorigenesis. For instance, miR-577 expression was revealed to be decreased in colorectal cancer samples and cell lines, and miR-577 overexpression inhibited tumor cell proliferation and metastasis (33,34). Furthermore, miR-577 inhibited the proliferation and EMT process via suppressing Rab25 expression in breast cancer (35). miR-577 may also suppress the progression of hepatocellular carcinoma, osteosarcoma and glioblastoma via targeting the Wnt/β-catenin signaling pathway (22,36,37). The present study revealed that the expression level of miR-577 was decreased in pancreatic cancer tissues and cells; additionally, miR-577 inhibition significantly enhanced proliferation, migration and invasion capabilities of pancreatic cancer cells, and miR-577 suppressed the activity of the Wnt/β-catenin signaling. Our data indicated that miR-577 is a tumor suppressor in pancreatic cancer.

The Wnt/β-catenin signaling pathway contributes to the progression of pancreatic cancer. It was reported that the Wnt signal inhibits the apoptosis of pancreatic cancer cells by increasing the expression of survivin, a member of apoptosis suppressors (38). Reportedly, SMARCAD1 promotes the growth and metastasis of pancreatic cancer by activating the Wnt/β-catenin pathway (39). In addition, the activation of the Wnt/β-catenin pathway has also been revealed to induce the resistance of pancreatic cancer cells to gemcitabine (40–42). In the present study, it was confirmed that miR-577 targets LPR6 and β-catenin in pancreatic cancer cells, and LPR6 and β-catenin expression levels were positively regulated by FGD5-AS1, which helps to clarify the mechanism of the activation of the Wnt/β-catenin pathway in pancreatic cancer.

lncRNAs may sponge miRNAs via miRNA response elements, and function as competing endogenous RNAs (ceRNAs), thereby indirectly modulating the translation of mRNAs (9). There are several studies suggesting that FGD5-AS1 functions as a ceRNA to regulate the biological behaviors of cancer cells. In particular, FGD5-AS1 facilitated non-small cell lung carcinoma cell proliferation via suppressing miR-107 to upregulate FGFRL1 (43); FGD5-AS1 facilitated oral squamous cell carcinoma progression by sponging miR-520b and inducing the expression of USP21 (44). The present study revealed that FGD5-AS1 was a ceRNA for miR-577, and it functioned as a molecular sponge to decoy miR-577; additionally, FGD5-AS1 knockdown in SW1990 cells suppressed the expression levels of the Wnt/β-catenin pathway-related proteins Axin2, c-Myc and cyclin D1. These results further indicated that the interaction between FGD5-AS1 and miR-577 was involved in the aberrant activation of the Wnt/β-catenin signaling in pancreatic cancer.

**Figure 7.**
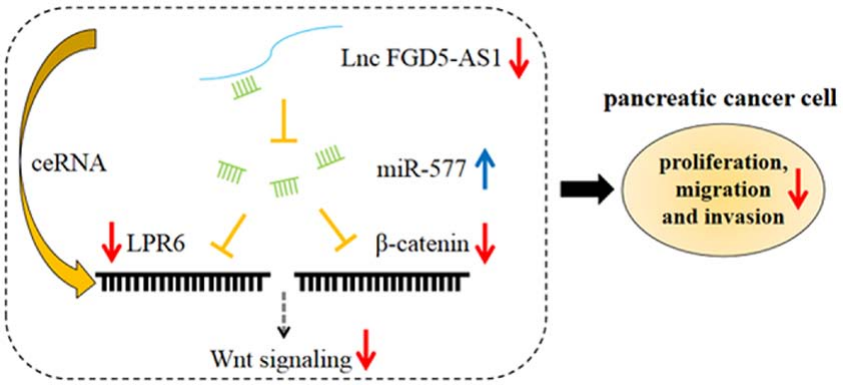
Graphical abstract. Long non-coding RNA FGD5-AS1 expression is markedly elevated in pancreatic cancer tissues and cells, and it could upregulate the expression of LPR6 and β-catenin by suppressing miR-577 expression via a ceRNA mechanism. Therefore, FGD5-AS1 modulated proliferation, migration and invasion of pancreatic cancer cells through miR-577/Wnt signaling axis. Lnc, long non-coding; miR, microRNA; ce, competing endogenous.

To sum up, in the present study, for the first time to the best of our knowledge, it was demonstrated that FGD5-AS1 was highly expressed in pancreatic cancer, and FGD5-AS1 promoted pancreatic cancer cell proliferation, migration and invasion. By contrast, miR-577 functioned as a tumor suppressor in pancreatic cancer, inhibiting cancer cell proliferation, migration and invasion. Additionally, FGD5-AS1 activated the Wnt/β-catenin signaling via suppressing miR-577 (Fig. 7). Our study helped to clarify the mechanism of pancreatic cancer progression and provided useful insights into the treatment of this disease. There are some limitations in the present study. The number of subjects was limited, and thus it was impossible to analyze the pathological characteristics and survival time more accurately. Therefore, whether FGD5-AS1 can be extended to a wider range of applications has yet to be determined. Notably, excluding functioning as a ceRNA, lncRNA FGD5-AS1 may exert its biological effect via functioning as a transcription regulator or protein-binding RNA, and whether FGD5-AS1 could regulate the progression of pancreatic cancer via these mechanisms requires further investigation in subsequent studies.

## Supplementary Material

Supporting Data

Supporting Data

Supporting Data

Supporting Data

## Figures and Tables

**Table I.** Primer sequences for RT-qPCR.

Genes	Primer sequences (5′-3′)
FGD5-AS1	F: AGAAGCGGAGGGGTGAAAAT
	R: CCGCCTTATAGTTGGCCCTC
LRP6	F: AGGCACTTACTTCCCTGCAA
	R: GGGCACAGGTTCTGAATCAT
β-catenin	F: GACATCAACGTGGTGACCTG
	R: GCTGGCTCTGTGATTTCCTC
GAPDH	F: GGAGCGAGATCCCTCCAAAAT
	R: GGCTGTTGTCATACTTCTCATGG
miR-577	F: TGCGGTAGATAAAATATTGG
	R: CCAGTGCAGGGTCCGAGGT
U6	F: GCTCGCTTCGGCAGCACA
	R: GAGGTATTCGCACCAGAGGA

RT-qPCR, reverse transcription quantitative polymerase chain reaction; LRP6, low-density lipoprotein receptor-related protein 6; GAPDH, glyceraldehyde-3-phosphate dehydrogenase; F, forward; R, reverse.

## Data Availability

The datasets used and/or analyzed during the current study are available from the corresponding author on reasonable request.

## References

[1] Klaiber U, Hackert T, Neoptolemos JP (2019). Adjuvant treatment for pancreatic cancer. Transl Gastroenterol Hepatol.

[2] Sung H, Ferlay J, Siegel RL, Laversanne M, Soerjomataram I, Jemal A, Bray F (2021). Global cancer statistics 2020: GLOBOCAN estimates of incidence and mortality worldwide for 36 cancers in 185 countries. CA Cancer J Clin.

[3] Kong K, Guo M, Liu Y, Zheng J (2020). Progress in animal models of pancreatic ductal adenocarcinoma. J Cancer.

[4] Ma YY, Shi JJ, Chen JB, Xu KC, Niu LZ (2020). Irreversible electroporation for liver metastasis from pancreatic cancer: A case report. World J Clin Cases.

[5] Perinel J, Adham M (2019). Palliative therapy in pancreatic cancer-palliative surgery. Transl Gastroenterol Hepatol.

[6] Liu G, Jiang Z, Qiao M, Wang F (2019). Lnc-GIHCG promotes cell proliferation and migration in gastric cancer through miR-1281 adsorption. Mol Genet Genomic Med.

[7] Fu R, Wang X, Hu Y, Du H, Dong B, Ao S, Zhang L, Sun Z, Zhang L, Lv G (2019). Solamargine inhibits gastric cancer progression by regulating the expression of lncNEAT1_2 via the MAPK signaling pathway. Int J Oncol.

[8] Evans JR, Feng FY, Chinnaiyan AM (2016). The bright side of dark matter: lncRNAs in cancer. J Clin Invest.

[9] Liu XH, Sun M, Nie FQ, Ge YB, Zhang EB, Yin DD, Kong R, Xia R, Lu KH, Li JH (2014). Lnc RNA HOTAIR functions as a competing endogenous RNA to regulate HER2 expression by sponging miR-331-3p in gastric cancer. Mol Cancer.

[10] Shen LS, Hu XF, Chen T, Shen GL, Cheng D (2019). Integrated network analysis to explore the key mRNAs and lncRNAs in acute myocardial infarction. Math Biosci Eng.

[11] Li S, Liu X, Li H, Pan H, Acharya A, Deng Y, Yu Y, Haak R, Schmidt J, Schmalz G (2018). Integrated analysis of long noncoding RNA-associated competing endogenous RNA network in periodontitis. J Periodontal Res.

[12] Lei Y, Shi Y, Duan J, Liu Y, Lv G, Shi R, Zhang F, Yang Q, Zhao W (2019). Identification of alternative splicing and lncRNA genes in pathogenesis of small cell lung cancer based on their RNA sequencing. Adv Clin Exp Med.

[13] Zhu H, Lu J, Zhao H, Chen Z, Cui Q, Lin Z, Wang X, Wang J, Dong H, Wang S (2018). Functional long noncoding RNAs (lncRNAs) in clear cell kidney carcinoma revealed by reconstruction and comprehensive analysis of the lncRNA-miRNA-mRNA regulatory network. Med Sci Monit.

[14] Hamilton MJ, Girke T, Martinez E (2018). Global isoform-specific transcript alterations and deregulated networks in clear cell renal cell carcinoma. Oncotarget.

[15] Li D, Jiang X, Zhang X, Cao G, Wang D, Chen Z (2019). Long noncoding RNA FGD5-AS1 promotes colorectal cancer cell proliferation, migration, and invasion through upregulating CDCA7 via sponging miR-302e. In Vitro Cell Dev Biol Anim.

[16] Fan J, Wei Q, Liao J, Zou Y, Song D, Xiong D, Ma C, Hu X, Qu X, Chen L (2017). Noncanonical Wnt signaling plays an important role in modulating canonical Wnt-regulated stemness, proliferation and terminal differentiation of hepatic progenitors. Oncotarget.

[17] Zhang L, Cheng H, Yue Y, Li S, Zhang D, He R (2018). H19 knockdown suppresses proliferation and induces apoptosis by regulating miR-148b/WNT/β-catenin in ox-LDL-stimulated vascular smooth muscle cells. J Biomed Sci.

[18] Li JH, Liu S, Zhou H, Qu LH, Yang JH (2014). starBase v2.0: Decoding miRNA-ceRNA, miRNA-ncRNA and protein-RNA interaction networks from large-scale CLIP-Seq data. Nucleic Acids Res.

[19] Livak KJ, Schmittgen TD (2001). Analysis of relative gene expression data using real-time quantitative PCR and the 2(−Delta Delta C(T)) Method. Methods.

[20] Zimmerman M, Hu X, Liu K (2010). Experimental metastasis and CTL adoptive transfer immunotherapy mouse model. J Vis Exp.

[21] Yang J, Cong X, Ren M, Sun H, Liu T, Chen G, Wang Q, Li Z, Yu S, Yang Q (2019). Circular RNA hsa_circRNA_0007334 is predicted to promote MMP7 and COL1A1 expression by functioning as a miRNA sponge in pancreatic ductal adenocarcinoma. J Oncol.

[22] Zhang W, Shen C, Li C, Yang G, Liu H, Chen X, Zhu D, Zou H, Zhen Y, Zhang D (2016). miR-577 inhibits glioblastoma tumor growth via the Wnt signaling pathway. Mol Carcinog.

[23] Ilic M, Ilic I (2016). Epidemiology of pancreatic cancer. World J Gastroenterol.

[24] Grant TJ, Hua K, Singh A (2016). Molecular pathogenesis of pancreatic cancer. Prog Mol Biol Transl Sci.

[25] Masiak-Segit W, Rawicz-Pruszyński K, Skórzewska M, Polkowski WP (2018). Surgical treatment of pancreatic cancer. Pol Przegl Chir.

[26] Lee YT, Tan YJ, Oon CE (2018). Molecular targeted therapy: Treating cancer with specificity. Eur J Pharmacol.

[27] Duguang L, Jin H, Xiaowei Q, Peng X, Xiaodong W, Zhennan L, Jianjun Q, Jie Y (2017). The involvement of lncRNAs in the development and progression of pancreatic cancer. Cancer Biol Ther.

[28] Chandra Gupta S, Nandan Tripathi Y (2017). Potential of long non-coding RNAs in cancer patients: From biomarkers to therapeutic targets. Int J Cancer.

[29] Li Y, Yang X, Kang X, Liu S (2019). The regulatory roles of long noncoding RNAs in the biological behavior of pancreatic cancer. Saudi J Gastroenterol.

[30] Deng SJ, Chen HY, Ye Z, Deng SC, Zhu S, Zeng Z, He C, Liu ML, Huang K, Zhong JX (2018). Hypoxia-induced LncRNA-BX111 promotes metastasis and progression of pancreatic cancer through regulating ZEB1 transcription. Oncogene.

[31] Fu Z, Chen C, Zhou Q, Wang Y, Zhao Y, Zhao X, Li W, Zheng S, Ye H, Wang L (2017). LncRNA HOTTIP modulates cancer stem cell properties in human pancreatic cancer by regulating HOXA9. Cancer Lett.

[32] Zhou L, Liang X, Zhang L, Yang L, Nagao N, Wu H, Liu C, Lin S, Cai G, Liu J (2016). MiR-27a-3p functions as an oncogene in gastric cancer by targeting BTG2. Oncotarget.

[33] Wang Y, Lu Z, Wang N, Feng J, Zhang J, Luan L, Zhao W, Zeng X (2018). Long noncoding RNA DANCR promotes colorectal cancer proliferation and metastasis via miR-577 sponging. Exp Mol Med.

[34] Du C, Wang HX, Chen P, Chen CH (2019). STAT3-induced upregulation of lncRNA DUXAP8 functions as ceRNA for miR-577 to promote the migration and invasion in colorectal cancer through the regulation of RAB14. Eur Rev Med Pharmacol Sci.

[35] Yin C, Mou Q, Pan X, Zhang G, Li H, Sun Y (2018). MiR-577 suppresses epithelial-mesenchymal transition and metastasis of breast cancer by targeting Rab25. Thorac Cancer.

[36] Wang LY, Li B, Jiang HH, Zhuang LW, Liu Y (2015). Inhibition effect of miR-577 on hepatocellular carcinoma cell growth via targeting β-catenin. Asian Pac J Trop Med.

[37] Jiang Z, Jiang C, Fang J (2018). Up-regulated lnc-SNHG1 contributes to osteosarcoma progression through sequestration of miR-577 and activation of WNT2B/Wnt/β-catenin pathway. Biochem Biophys Res Commun.

[38] Modi S, Kir D, Banerjee S, Saluja A (2016). Control of apoptosis in treatment and biology of pancreatic cancer. J Cell Biochem.

[39] Liu F, Xia Z, Zhang M, Ding J, Feng Y, Wu J, Dong Y, Gao W, Han Z, Liu Y (2019). SMARCAD1 promotes pancreatic cancer cell growth and metastasis through Wnt/β-catenin-mediated EMT. Int J Biol Sci.

[40] Nagano H, Tomimaru Y, Eguchi H, Hama N, Wada H, Kawamoto K, Kobayashi S, Mori M, Doki Y (2013). MicroRNA-29a induces resistance to gemcitabine through the Wnt/β-catenin signaling pathway in pancreatic cancer cells. Int J Oncol.

[41] Zhan T, Chen X, Tian X, Han Z, Liu M, Zou Y, Huang S, Chen A, Cheng X, Deng J (2020). MiR-331-3p links to drug resistance of pancreatic cancer cells by activating WNT/β-catenin signal via ST7L. Technol Cancer Res Treat.

[42] Zhou C, Yi C, Yi Y, Qin W, Yan Y, Dong X, Zhang X, Huang Y, Zhang R, Wei J (2020). LncRNA PVT1 promotes gemcitabine resistance of pancreatic cancer via activating Wnt/β-catenin and autophagy pathway through modulating the miR-619-5p/Pygo2 and miR-619-5p/ATG14 axes. Mol Cancer.

[43] Fan Y, Li H, Yu Z, Dong W, Cui X, Ma J, Li S (2020). Long non-coding RNA FGD5-AS1 promotes non-small cell lung cancer cell proliferation through sponging hsa-miR-107 to up-regulate FGFRL1. Biosci Rep.

[44] Liu L, Zhan Y, Huang Y, Huang L (2020). LncRNA FGD5-AS1 can be predicted as therapeutic target in oral cancer. J Oral Pathol Med.

