# Supplementary material for: FGD5-AS1 is an oncogenic lncRNA in pancreatic cancer and regulates the Wnt/β-catenin signaling pathway via miR-577
Source: Oncol Rep. 2021 Nov 24;47(1):21. doi: 10.3892/or.2021.8232 (PMC8630524; doi:10.3892/or.2021.8232)

Figure S1. Both starBase database and LncBase Predict v.2 database predicted that miR-577 was a target of FGD5-AS1. Lnc, long non-coding; miR, microRNA.

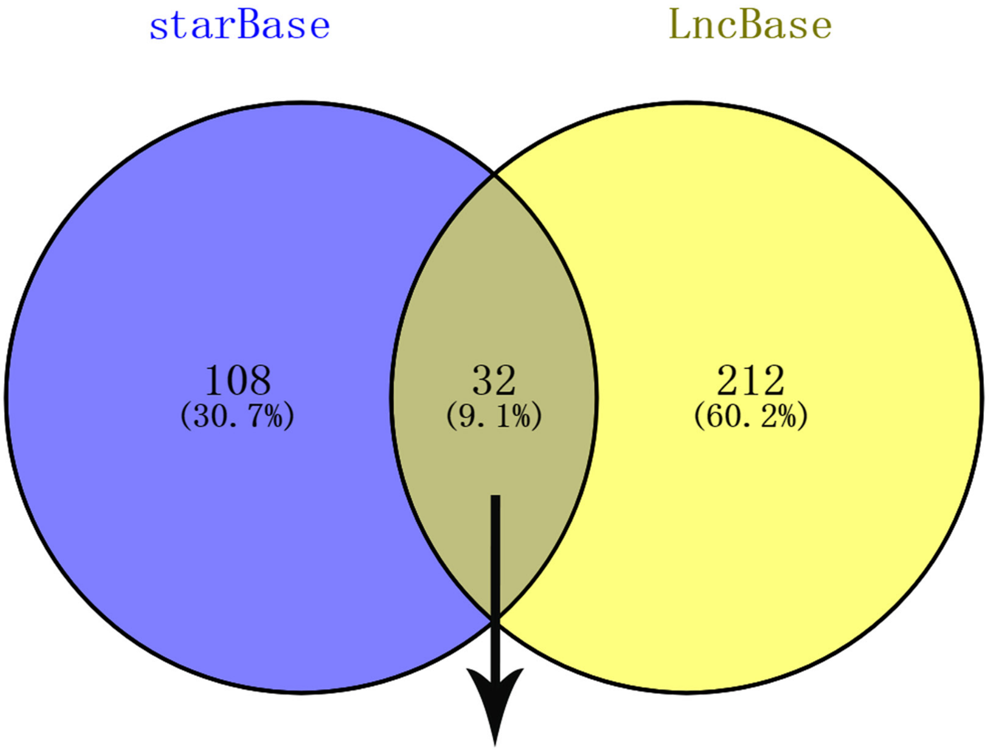


**hsa-miR-380-3p、hsa-miR-577、hsa-miR-3163、hsa-miR-944、hsa-miR-126-5p、hsa-miR-520d-5p、hsa-miR-524-5p、hsa-miR-3613-5p、hsa-miR-493-5p、hsa-miR-323a-3p、hsa-miR-374c-3p、hsa-miR-1179、hsa-miR-421、hsa-miR-512-3p、hsa-miR-506-3p、hsa-miR-1323、hsa-miR-548o-3p、hsa-miR-129-5p、hsa-miR-106a-5p、hsa-miR-670-3p、hsa-miR-320d、hsa-miR-4429、hsa-miR-320c、hsa-miR-320a、hsa-miR-320b、hsa-miR-6835-3p、hsa-miR-7853-5p、hsa-miR-105-5p、hsa-miR-33a-5p、hsa-miR-885-5p、hsa-miR-374b-3p、hsa-miR-153-3p**

Figure S2. The expression levels of miR-577 in SW1990 cells transfected with miR-577 mimic. ***P<0.001. miR, microRNA.

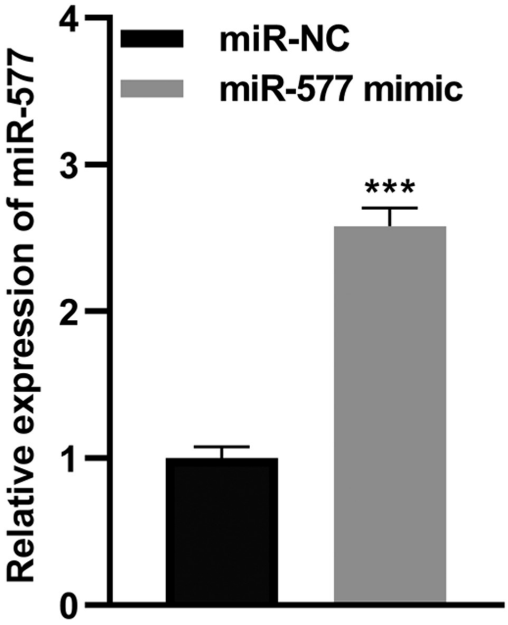

Figure S3. FGD5-AS1 regulates the metastasis of SW1990 cells *in vivo*, which was evaluated by hematoxylin and eosin staining. si-, small interfering.

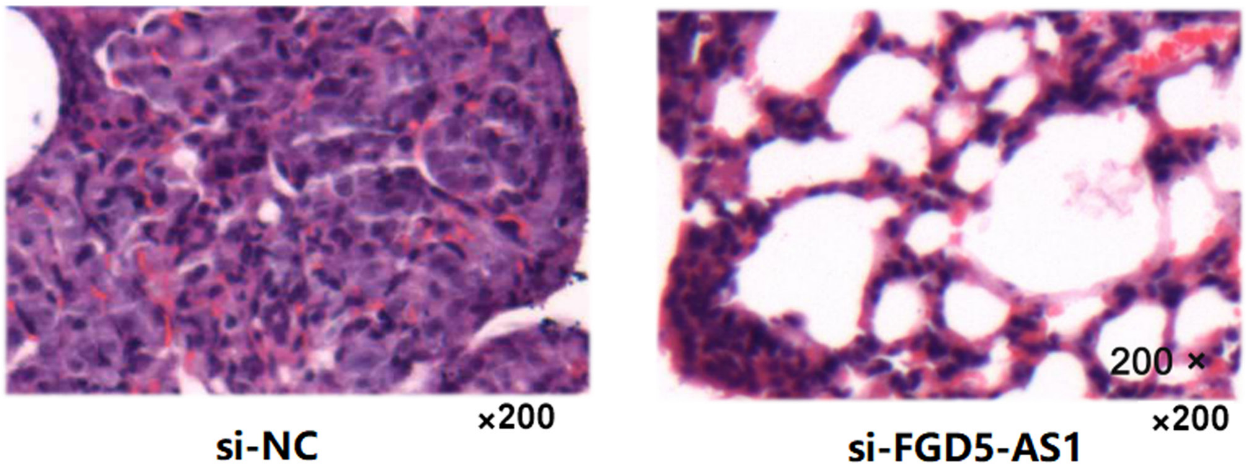

Supplement: Supporting Data [file Supplementary_Data1.pdf]
