# Supplementary material for: FGD5-AS1 is an oncogenic lncRNA in pancreatic cancer and regulates the Wnt/β-catenin signaling pathway via miR-577
Source: Oncol Rep. 2021 Nov 24;47(1):21. doi: 10.3892/or.2021.8232 (PMC8630524; doi:10.3892/or.2021.8232)
Supplement: Supporting Data [file Supplementary_Data4.pdf]

Table SIII. Pathological evaluation of the severity of lung metastasis of SW1990 cells in nude mice.

Lung metastasis	Severe	Moderate	Mild	P-value
si-NC group (n=10)	6	3	1	0.016
si-FGD5-AS1 group (n=10)	1	2	7	

si-, small interfering; NC, negative control.
